# Ultrasound Treatment on Stability of Total and Individual Anthocyanin Extraction from Blueberry Pomace: Optimization and Comparison

**DOI:** 10.3390/molecules24142621

**Published:** 2019-07-18

**Authors:** Weiwei Hu, Hui Gong, Lanqi Li, Shiguo Chen, Xingqian Ye

**Affiliations:** Zhejiang Key Laboratory for Agro-Food Processing, Fuli Institute of Food Science, College of Biosystem Engineering and Food Science, Zhejiang University, Hangzhou 310058, China

**Keywords:** pomace, anthocyanin, identification, ultrasound-assisted extraction, conventional solvent extraction, comparison

## Abstract

Blueberry pomace is abundant in anthocyanins. This work characterized the anthocyanins in blueberry pomace, discussed the stability of anthocyanins under ultrasound treatment, and compared the extraction conditions for different anthocyanin compositions. Thirteen anthocyanins were identified, and malvidin-3-galactoside (18.56%), which represented the most abundant anthocyanin, was selected as the individual analyte. The general linear model univariate analysis revealed that ultrasound-assisted extraction (UAE) resulted in higher recoveries of both total anthocyanins (TA) and individual anthocyanins (IA) when compared with conventional solvent extraction. The optimized extraction conditions for TA and IA were UAE in pure methanol (12.49 mg/g dry weight) at 25 °C for 30 min and UAE in 70% ethanol (3.57 mg/g dry weight) at 40 °C for 40 min, respectively. Moreover, IA was more vulnerable to degradation compared with TA. Therefore, a specific extraction process of IA is significant for monomer preparation, and harsh conditions should be avoided in UAE.

## 1. Introduction

Blueberries (*Vaccinium* spp.) are widely cultivated in many countries around the world and have become increasingly popular owing to their high nutritional value and tasty flavor [1]. The fruit contains appreciable levels of plant constituents, including phenolic compounds, anthocyanins, procyanidins, chlorogenic acid, and flavanols, which have biological activity and may provide health benefits (e.g., decrease blood pressure, cholesterol, and bone protection) as dietary antioxidants [2,3]. However, due to the limited shelf life of fresh berries, the fruit is commonly prepared as processed products, such as juice, wine, jam, and marmalade [4,5]. Supposing that the total blueberry production in 2017 was processed in Canada, the second largest producer worldwide after the United States reached 176,641 tons with some consumed fresh and some being processed [5]. Accordingly, large quantities of pomace generated through berry processing represent an excellent source of phenolic compounds and could be used as polyphenol-rich materials. Anthocyanins, which are phenolic compounds belonging to the family of flavonoids that constitute the largest group of water-soluble pigments in the plant kingdom, are abundant in blueberry pomace and contribute to its red, purple, and blue hues [6]. As a consequence of the social trend toward avoiding the consumption of synthetic food components, anthocyanins have been considered as a natural alternative in the food industry. Moreover, the long-term consumption of anthocyanins reveals promising protective effects against several chronic diseases, such as cardiovascular disorders, neurodegenerative diseases, and cancer [5,7,8]. Thus, extracting valuable bioactive phytochemicals (e.g., anthocyanins) from the blueberry pomace for healthcare and lifestyle applications in foods, pharmaceuticals, or fine chemicals is an efficient way to maximize the valorization of this by-product.

A holistic approach (termed the “5-Stage Universal Recovery Process”) was designed to recover valuable compounds from natural sources such as blueberries. It is based on the stages of (i) macroscopic pre-treatment, (ii) separation of macro- and micro-molecules, (iii) extraction, (iv) purification, and (v) product formation [9]. Particularly, extraction, which is defined as a mass transport phenomenon in which insoluble solids (contained in a matrix) migrate into a solvent brought into content with the matrix, is the most important step in the downstream processing. Multi-factors including concentration gradient, diffusion coefficients, or boundary layer influence the mass transport phenomena. Heating, which increases mass transfer to a certain extent, also affects the extraction efficiency, as it can deteriorate the matrix or diminish the functionality of targeted compounds when high temperatures are used [10,11,12].

Various technologies recommended for the isolation of anthocyanins from natural sources employ organic solvents, supercritical fluid, or enzyme-assisted extraction methods [13,14,15], yet environmental impact, scalability, and production costs often inhibit the application of these procedures in the food industry.

Recently, new technologies that can reduce extraction time and solvent consumption have been developed for efficient extraction of anthocyanins compounds from plants. Garcia-Mendoza et al. [16] highlighted pressurized liquid extraction to efficiently obtain anthocyanins from juçara (*Euterpe edulis* Mart.) residue. Different solvents at 40, 60, and 80 °C were optimized, and the results indicated that pressurized liquid extraction provided anthocyanin-rich extracts when acidified solvents were used. Romero Diez et al. [17] discussed enhancing the extraction of anthocyanins from different wine lees by microwave pre-treatments by examining the influence of the parameters (temperature, solid–liquid ratio, and type of solvent) and the extraction kinetics in conventional solid–liquid extraction. It was demonstrated that microwave pre-treatment significantly improved anthocyanin extraction by up to two-fold and reduced the extraction time from 15 min to 90 s. Meanwhile, high hydrostatic pressure has also been emphasized as a potential application for anthocyanin extraction [18,19].

Alternatively, ultrasound, an oscillating sound pressure wave with a frequency over 20 kHz, has been shown to facilitate extraction by increasing the mass transfer between the solvent and the plant material [20]. As a green, non-thermal processing technique, ultrasound has been perceived as a more sustainable, green, and efficient solution for industrial application than conventional approaches, and its use in anthocyanin extraction has already been demonstrated. For instance, Backes et al. [21] compared three different techniques (heat, ultrasound, and microwave) for anthocyanin extraction maximization using the response surface methodology with three factors and five levels per factor according to a circumscribed central composite design. The results showed that ultrasound-assisted extraction (UAE) with the optimal extraction conditions of 310 W, 100% ethanol (EtOH), and 21 min was the most effective method. Pinela et al. [22] optimized heat-assisted extraction and UAE of anthocyanins from *Hibiscus sabdariffa* calyces as natural food colorants, and the achieved experimental data confirmed that UAE was the most efficient method, yielding 51.76 ± 3.70 mg anthocyanins/g extract. UAE has also been applied to recover the anthocyanins from purple sweet potato [23] and blueberry (*Vaccinium* ashei) wine pomace [24]. Considering that anthocyanins degrade under harsh conditions and are incompletely extracted under mild conditions, identifying the appropriate parameters for anthocyanin recovery is valuable. As part of ongoing work, the objective of this study was targeted on the most abundant and known structure individual anthocyanin (malvidin-3-galactoside) to investigate the effects of selected ultrasound parameters on its content changes vs. total anthocyanins changes. As far as the authors are aware, few studies refer to the stability of target anthocyanins under ultrasound treatment. Moreover, blueberry anthocyanins differ according to fruit variety, maturity, and cultivation region. A specific anthocyanin with its typical characteristics accounts for diverse benefits. Thus, the identification of anthocyanins in blueberries is fundamental in the development and the utilization of the fruit. 

In this work, extractions of the total anthocyanins (TA) and an individual anthocyanin (IA) from blueberry pomace by ultrasound and conventional solvent extraction treatment were systematically studied and compared, and the ultrasound effects on anthocyanin stability were also discussed. First, the composition and the concentration of anthocyanins in blueberry pomace were analyzed by liquid chromatography-mass spectrometry (LC–MS) and high performance liquid chromatography (HPLC) to assess the fingerprint information of the analytes. Then, the time-dependent anthocyanin contents were evaluated by conventional solvent extraction. Afterwards, the recovery of TA and IA by UAE was optimized via full factorial design and a general linear model (GLM) univariate algorithm and compared with conventional solvent extraction. Finally, optimal extraction conditions for different anthocyanin compositions were compared and discussed.

## 2. Results

### 2.1. Anthocyanin Composition of Blueberry Pomace

**Figure 1:**
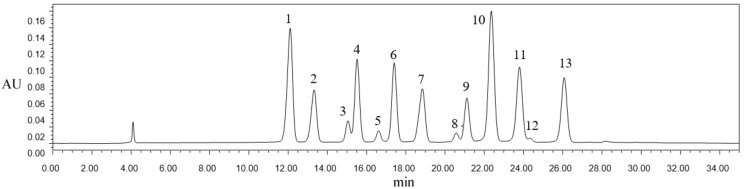
High performance liquid chromatography (HPLC) chromatogram of anthocyanins in blueberry pomace.

The anthocyanins in blueberry pomace were identified by LC–MS. Blueberry pomace extracts of conventional solvent extraction method were used for anthocyanins composition analysis. Figure 1 demonstrates the HPLC chromatogram of the anthocyanins, and the corresponding mass spectrum information, including peak number, retention time, molecular ions, formula, and proportion, is summarized in Table 1. Anthocyanins are a combination of carbon, hydrogen, and oxygen. Due to the inexact integral mass of hydrogen and oxygen, anthocyanins with unique formulas have unique masses. Hence, with highly accurate mass measurements, the species can be positively identified [25]. Herein, a total of 13 anthocyanins were detected in the blueberry extracts. Except for three unrecognizable peaks, other anthocyanins were monoglucoside, monogalactoside, or monoarabinoside derivatives of five anthocyanidins: delphinidin, cyanidin, petunidin, peonidin, and malvidin. Specifically, peak 2 and peak 4 had the same molecular ion at 465 m/z but different retention times (13.327 and 15.521 min, respectively). It was reported that the elution sequence of substances was positively correlated to the polarity when reversed-phase chromatography was applied. Accordingly, peak 2 and peak 4 were assigned to delphinidin-3-galactoside and delphinidin-3-glucoside, respectively. Similarly, peak 7 (cyanidin-3-glucoside) and peak 11 (petunidin-3-arabinoside) of the same [M+H]^+^ m/z 449 were identified according to Barnes et al. [25]. Petunidin-3-galactoside (peak 8) and petunidin-3-glucoside (peak 9) were also detected [26], along with malvidin-3-galactoside (peak 10) and malvidin-3-glucoside (peak 12) [27]. Moreover, malvidin (27.71%) and delphinidin (26.3%) glycosides constituted the major anthocyanins, which constituted more than half of the TA content, and malvidin-3-galactoside (peak 10; 18.56%) represented the most abundant IA. Li et al. [28] identified and quantified anthocyanins in 17 samples of blueberries of different varieties and geographical origins in China (e.g., Dandong (124°23′ E, 40°07′ N), Zhuanghe (122°30′ E, 39°75′ N), Suizhou (113°01′ E, 31°62′ N), and Qingdao (120°75′ E, 36°15′ N)], and their work revealed that the anthocyanin profiles from blueberries of all cultivars were similar, but the proportions were cultivar-dependent, although malvidin (41.0%), delphinidin (33.1%), and petunidin (17.3%) were the major contributors to the TA content. Therefore, malvidin-3-galactoside, which belongs to the malvidin family and presents the most abundant and known structure individual anthocyanin, was selected as the model IA analyte in the following ultrasound treatment.

### 2.2. Time-Dependent Anthocyanin Contents by Conventional Solvent Extraction

Figure S1 depicts the effect of time on the TA extracted by conventional solvent extraction. It was evident that the amount of TA extracted from blueberry pomace increased with extraction time, and the anthocyanin content nearly doubled after soaking for 3 h, whereas no significant increase was observed when the extraction time was prolonged further. Thus, 3 h extraction by conventional solvent extraction was selected as the control (CK) for comparison with UAE.

### 2.3. Effect of Ultrasound Time on the Stability of Total and Individual Anthocyanins

Figure S2 and Table S1 show the effect of ultrasound time on the amount of TA obtained from freeze-dried blueberry pomace at different time intervals during the UAE and conventional solvent extraction (control) using different temperatures and solvents. Generally, TA first increased and then decreased as the reaction time was prolonged, and a greater TA was recovered by UAE than conventional solvent extraction (*p* < 0.05). The TA contents recovered by UAE and conventional solvent extraction were significantly affected by the extraction time. Specifically, the lowest TA contents were observed at 5 and 50 min using aqueous and pure solvents, while the maximal TA was achieved at 10 min for 70% MeOH and 70% EtOH and at 20 min for 40 °C, 30 min for 10 and 25 °C of pure MeOH and pure ETOH, respectively. The amount of TA recovered by UAE reached saturation at around 10–30 min, and instead of increasing, the values decreased significantly after that, irrespective of the solvent used. As an example, for 70% EtOH, the TA amounts recovered by UAE at 10, 25, and 40 °C for 50 min were 6.08, 7.36, and 6.77% less, respectively, when compared with those at 10 min under the same condition. For 70% MeOH, the differences were 6.78, 3.40, and 6.13% between ultrasound for 50 and 10 min, and for the pure solvents, the TA contents obtained at 10, 25, and 40 °C were, respectively, 2.8, 6.79, and 18.40% (pure MeOH) and nearly 2.1, 4.3, and 9.0% (pure EtOH) less when UAE was applied for 50 vs. 20 min. The phenomenon can be explained because a short extraction time would lead to incomplete extraction, while an extended extraction would cause degradation of the TA in blueberry pomace. Moreover, the ultrasound time for maximum TA values depended on the solvent used. For 70% MeOH and 70% EtOH, the TA reached maximal values at 10 min at 10, 20, and 40 °C, indicating that the aqueous mixtures were preferred over the pure solvents for anthocyanin recovery by UAE, which is consistent with the observations recorded by Cai et al. [29] and Li et al. [30]. However, the data comparison presented in Table S1 shows that the TA extraction was maximal (12.49 mg/g) for pure MeOH at 25 °C when ultrasound was applied for 30 min. This observation may be related to the difference in polarity, viscosity, surface tension, and saturated vapor pressure between the four solvents.

**Figure 2:**
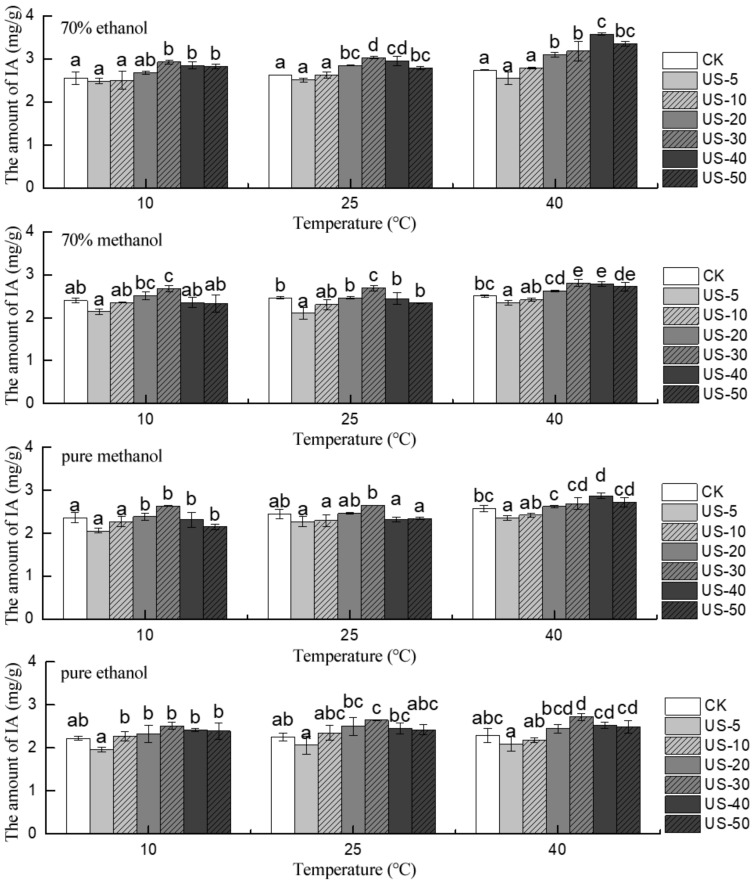
Effect of time on the stability of individual anthocyanin in blueberry pomace under ultrasound treatment and conventional solvent extraction. Different letters on bars show significant differences (*p* < 0.05).

For IA extraction, malvidin-3-galactoside, which represented the major anthocyanin present in the blueberry pomace, was selected as the target analyte. As shown in Figure 2 and Table S2, ultrasound treatments using different temperatures and solvents were compared with conventional solvent extraction (control). The largest amount of IA obtained by ultrasound was more than that by conventional solvent extraction (*p* < 0.05), and the IA content first increased and then decreased with the increase in reaction time. Generally, for 70% MeOH, 70% EtOH, pure MeOH, and pure EtOH, the IA content was lowest at 5 and 50 min and reached saturation at around 30–40 min of UAE. For 70% EtOH, the IA content obtained by UAE at 10, 25, and 40 °C was approximately 3.75, 5.42, and 5.88% lower at 50 than 30 min, and in 70% MeOH system, the same pattern was seen with 13.05%, 13.01%, and 3.20% less at 50 min than at 30 min under the same condition. This trend was consistent for pure MeOH and pure EtOH with decreases of 18.25, 11.69, and 5.57% and 4.78, 8.30, and 8.52% at 10, 25, and 40 °C, respectively, between UAE for 50 and 10 min. According to the comparison of the total data in Table S2, the optimized ultrasound condition for IA extraction from blueberry pomace was 70% EtOH at 40 °C for 40 min, and the maximum value was 3.57 mg/g.

### 2.4. Effect of Ultrasound Temperature on the Stability of Total and Individual Anthocyanins

Figure S3 illustrates the effect of ultrasound temperature on the stability of the TA exposed to UAE and conventional solvent extraction (control) for different time intervals using different solvents. Considering that the largest values of TA and IA were observed when UAE was applied for 10 min (aqueous solvents) and 30 min (pure solvents), these two time-points (10 and 30 min) were selected as the reaction times. For both the control and the UAE groups with different solvents, due to the promoted dissolution, the TA contents increased gradually with the increasing temperature. Nevertheless, the cavitation effect is reported to decrease with the increase of temperature; that is, the extraction amounts at high temperature should be lower than those at low temperature because of the accompanying cavitation-induced damage. Though the intensity of ultrasound cavitation is weaker at high temperature, the damaging effect reduces as well. Accordingly, in a certain temperature range, the amount of anthocyanin extracted by ultrasound will not necessarily decrease with the increase of temperature and might even be enhanced. When assessing the effects of the extraction methods and conditions on anthocyanin compositions, Wang et al. [6] showed that, as the extraction temperature was increased from 50 to 60 and 70 °C, the extraction amount of blueberry anthocyanins in MeOH was also increased. In the current work, the extraction of the TA from blueberry pomace also increased when the temperature was increased (10 °C < 25 °C < 40 °C). When UAE was applied for 10 min, the TA contents at 10 °C were 94.8, 95.5, 92.1, and 93.8% of those at 40 °C when using the solvents 70% EtOH, 70% MeOH, pure MeOH, and pure EtOH, respectively. When UAE was applied for 30 min, the corresponding values changed to 96.7, 94.7, 89.4, and 93.9% of the TA contents at 40 °C using the same solvents. Meanwhile, applying UAE for 10 min revealed that the biggest difference between the ultrasound and the control groups was for 70% MeOH, and the smallest difference was for 70% MeOH and pure EtOH. For 30 min of UAE, the biggest and the smallest differences were found in EtOH and 70% EtOH solvents, respectively.

**Figure 3:**
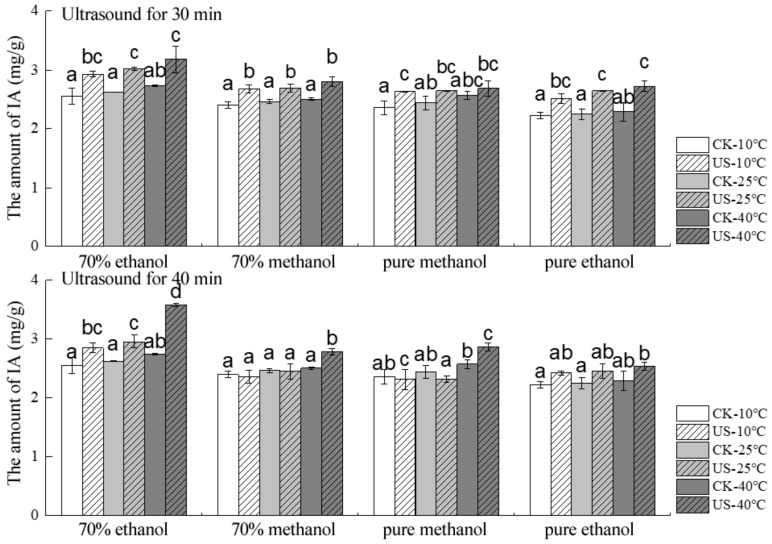
Effect of temperature on the stability of individual anthocyanin in blueberry pomace under ultrasound treatment and conventional solvent extraction. Different letters on bars show significant differences (*p* < 0.05).

Similarly, 30 and 40 min were selected as the reaction times to analyze the effect of ultrasound temperature on the stability of the IA contents, with malvidin-3-galactoside as the target analyte. As seen in Figure 3, for both the control and the UAE groups with different solvents, due to the promoted dissolution, the IA content increased gradually with the increasing temperature. It is likely that at high temperature, the intensity of the ultrasound cavitation is weaker, and so too is the damaging effect, whereas solubilization will be promoted. Thus, at a certain temperature range, the amount of anthocyanin extracted by ultrasound will not necessarily decrease with the increase of temperature. However, if the temperature rises too high, the amount of extraction will decrease, as thermal degradation will be introduced. Overall, the extraction of the IA from blueberry pomace increased when a higher temperature was used (10 °C < 25 °C < 40 °C). After UAE for 30 min, the IA contents of blueberry pomace extracted at 10 °C were only 92.13, 95.37, 97.76, and 92.27% of those at 40 °C for 70% EtOH, 70% MeOH, pure MeOH, and pure EtOH, respectively. After ultrasound treatment at 10 °C for 40 min, the corresponding values changed to 79.83, 84.53, 80.48, and 95.65% of the IA content at 40 °C using the same solvents. At the same time, pure EtOH showed the biggest difference in the IA contents between the ultrasound and the control groups, and pure MeOH displayed the smallest difference, respectively, for 30 min UAE. Similarly, when ultrasound was applied for 40 min, 70% EtOH was the solvent that showed the biggest difference between the ultrasound and the control groups, and pure MeOH presented the smallest difference between these groups.

### 2.5. Effect of Ultrasound Solvent on the Stability of Total and Individual Anthocyanins

Figure S4 reveals the effect of solvent on the amount of TA obtained at different time intervals by UAE and conventional solvent extraction (control) using different ultrasound temperature and solvents (70% EtOH, 70% MeOH, pure MeOH, and pure EtOH). It was evident that the peak value of TA extracted from blueberry pomace by ultrasound in different solvents was significantly higher than that of the control. More specifically, the highest amounts of TA obtained at 10 °C for 70% EtOH, 70% MeOH, pure MeOH, and pure EtOH were 3.18, 5.48, 11.71, and 4.03% higher, respectively, than those of the control. The peak values of TA extracted by ultrasound at 25 °C were 4.37, 3.82, 23.09, and 5.27% higher, and at 40 °C, they were 4.49, 6.57, 21.58, and 6.48% higher for 70% EtOH, 70% MeOH, pure MeOH, and pure EtOH, respectively, compared with conventional solvent extraction. When comparing the ultrasound treatments, there were significant differences in the amounts of TA extracted between 10 and 30 min under different temperatures and solvents (*p* < 0.05).

**Figure 4:**
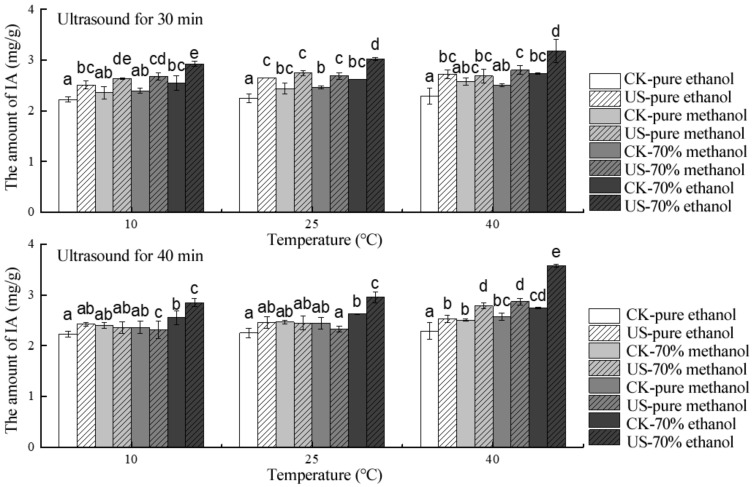
Effect of solvent on the stability of individual anthocyanin in blueberry pomace under ultrasound treatment and conventional solvent extraction. Different letters on bars show significant differences (*p* < 0.05).

For IA (malvidin-3-galactoside) extraction from blueberry pomace by ultrasound in different solvents, the peak value was significantly higher than that of the control group (Figure 4). At 10 °C, the IA contents attained by conventional solvent extraction in 70% EtOH, 70% MeOH, pure MeOH, and pure EtOH only accounted for 87.03, 89.55, 89.73, and 92.14% of the IA extracted by ultrasound. The maximal IA contents extracted by conventional solvent extraction in 70% EtOH, 70% MeOH, pure MeOH, and pure EtOH system at 25 °C were nearly 86.75, 91.44, 92.07, and 84.90% of that obtained by ultrasound, and the corresponding values at 40 °C were approximately 76.75, 88.96, 89.54, and 84.19%, respectively. Moreover, more IA content could be extracted by 70% EtOH than any of the other solvents among the different temperatures, and the extraction of the IA was significantly affected by the ultrasound solvent (*p* < 0.05).

### 2.6. Ultrasound Interactions on Total and Individual Anthocyanins Extraction

**Figure 5:**
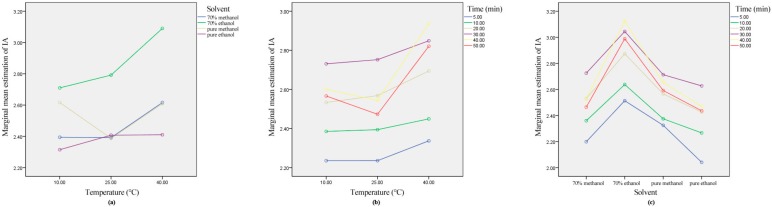
Effect of interaction on individual anthocyanins from blueberry pomace during ultrasound extraction course. Letters (**a**–**c**) represent interactions of solvent–temperature, temperature–time, and solvent–time.

A general linear model (GLM) univariate method with all factor models was employed to analyze the main effects and the interactions of the three factors on the TA recovered by UAE. For the ultrasound interactions, there was no significant effect on TA extraction for the temperature–time–solvent interactions. However, the interactions of any two factors, including solvent–temperature, temperature–time, and solvent–time, revealed a significant effect on TA extraction (*p* < 0.05). Moreover, the map of edge mean estimation represents the interaction of the factors, which provides an intuitive understanding of the interaction effects. As evidenced in Figure S5, the mean value lines of the solvent–temperature, the temperature–time, and the solvent–time interactions were not parallel, which further confirmed that the interactions between any two factors of solvent, temperature, and time significantly impacted the TA extraction (*p* < 0.05). For the ultrasound interactions on IA (malvidin-3-galactoside) extraction, the GLM univariate method with all factor models and edge mean estimation maps was also used for significance analysis. As shown in Figure 5, the same results as mentioned above for TA were obtained. Namely, unlike the temperature–time–solvent interactions, IA extraction was significantly affected by the interactions between any two factors, including solvent–temperature, temperature–time, and solvent–time (*p* < 0.05).

## 3. Discussion

In this work, TA and IA extractions from blueberry pomace by UAE and conventional solvent extraction were systematically studied. Malvidin-3-galactoside, which belongs to the malvidin family and presents the most abundant and known structure individual anthocyanin, was selected as the target IA by UAE. Similarities and differences were obtained in TA and IA amounts extracted when comparing the two treatments. Regarding the similarities, the amounts of TA and IA extracted from blueberry pomace first increased and then decreased as the ultrasound time increased (0–50 min), and the time for maximal anthocyanin contents varied among the different solvents. The extraction of TA and IA at the three temperatures was investigated, and the results revealed that more extracts could be obtained at relatively higher temperatures. These findings were consistent with previous reported studies [6]. However, thermal degradation would have been introduced if an over-high extraction temperature had been employed in the ultrasound treatment. Ekici et al. [31] examined the stability changes of anthocyanins in black carrot, red cabbage, and grape peel at pH 3–7 at 70–90 °C for 0–120 min, and the work concluded that the anthocyanins in all three materials decreased with the increasing of temperature when pH and time were unchanged. For example, his group researched that, when the black carrot was treated at pH 3 for 30 min, the anthocyanin contents were lowered by 4.02, 7.22, and 30.02% at 70, 80, and 90 °C, respectively. Besides, there were significant differences between the control and the ultrasound groups based on marginal mean estimation and one-way analysis of variance, and it was found that the peak values of TA and IA extracted by ultrasound in different solvents were significantly larger than those obtained by conventional solvent extraction. Moreover, unlike the temperature–time–solvent interactions, the interactions of any two factors, including solvent–temperature, temperature–time, and solvent–time, significantly affected TA and IA amounts extracted (*p* < 0.05). 

Regarding the differences among the solvents investigated, the maximal values achieved in 30 min by ultrasound were 70% EtOH < 70% MeOH < pure EtOH < pure MeOH for TA and pure EtOH < pure MeOH < 70% MeOH < 70% EtOH for IA. More importantly, it was found that the interaction of solvent, temperature, and time had no significant effect on TA extraction, while it had significant effect on IA (*p* < 0.05), and the degradation extent of single anthocyanin in blueberry pomace was larger than that of total anthocyanin after extraction saturation. The individual anthocyanin (IA) was seemingly more vulnerable to degradation compared with TA during UAE. The optimum solvent for ultrasound extraction of TA was pure MeOH followed by 70% MeOH, while the optimum solvent for ultrasound extraction of IA was 70% EtOH followed by pure MeOH. These conclusions were similar to previously published works. Cai et al. [29] compared the extraction efficiency of anthocyanins from purple sweet potatoes using conventional extraction (CE), ultrasound-assisted extraction (UAE), and another accelerated-solvent extraction with different temperature (60, 70, and 80 °C), extraction time (90, 120, and 150 min), and aqueous ethanol (80%, 90%, and 100%, *v/v*). Their work demonstrated that 80% (*v/v*) ethanol was optimized for theoretical maximum anthocyanin yield in CE treatment, while in UAE, the concentration raised to 90% ethanol (*v/v*). Overall, the optimized extraction condition for TA was ultrasound for 30 min at 25 °C in pure MeOH with the maximum value of 12.49 mg/g (dry weight), and for IA, the optimal condition was ultrasound for 40 min at 40 °C in 70% EtOH with the maximum value of 3.57 mg/g (dry weight). 

Moreover, for the industrial level, the extraction process of IA from blueberry pomace needs to be optimized separately from the TA, as the concrete contents under different cases will be expanded with thousands of increased raw materials in practical industry application, generating significant differences from a practical point of view. Therefore, the study on the separation and the purification process of IA has more value and significance for the technical reference of large-scale production of anthocyanin monomers.

## 4. Materials and Methods 

### 4.1. Materials

Blueberries (*Vaccinium* spp.) were purchased from Shengzhou (Zhejiang, China). Methanol (MeOH), acetonitrile (ACN), and methane acid were of HPLC grade and were obtained from Aladdin (Shanghai, China). Sodium acetate trihydrate, KCl, HCl, EtOH, and all other chemicals were of analytical grade and were used without further purification.

### 4.2. Blueberry Ppomace Samples

In this work, fresh blueberries were treated with a juicer (mechanical force) to mimic the processing of crushing, pressing, and filtration of industrial juice production. After treatment, the juice was filtered, and the remaining blueberry pomace from the juicer was collected as pomace samples. The resultant blueberry pomace was freeze-dried using an Alpha 1-4 LD lyophiliser (Martin Christ, Osterode, Germany) at –50 °C for about 48 h. The freeze-dried powder was milled into particles smaller than 0.180 mm (No. 80 mesh), sealed, and stored at 4 °C.

### 4.3. Identification of Anthocyanins in Blueberry Pomace

Blueberry pomace extracts of the conventional solvent extraction method were analyzed for anthocyanins composition. The LC–MS chromatography system consisted of an Agilent 1100 liquid chromatograph equipped with an Agilent C18 column packed with Zorbax (4.6 × 250 mm, 5 μm; Agilent Technologies Co., Ltd., Shanghai, China), an autosampler module, a degasser, a binary pump, a column heater/selector, a UV–visible-diode array detector from Agilent Technologies (Santa Clara, CA, USA), and a quadrupole mass spectrometer. Chromatographic separation was achieved using a gradient elution at 35 °C with aqueous formic acid (6% *v/v*; mobile phase A) and 6% *v/v* ACN–formic acid (mobile phase B) at a flow rate of 0.6 mL/min according to the following program: 85–70% B (0–20 min), 70–65% B (20–25 min), 65–85% B (25–30 min), and 85% B (30–35 min). Anthocyanins were detected at 520 nm. For mass spectra (MS) detection, nitrogen was used at a flow rate of 10 mL/min and a pressure of 30 psi, both as the drying and the nebulizing gas. The capillary voltage was set at 3500 V, and the temperature was held at 350 °C. The electrospray ionization source was operated in the positive ionization mode and scanned in the m/z range of 100–600. The secondary tandem mass spectrometry was performed at 100–500 m/z at a collision energy of 60 eV.

### 4.4. Ultrasound Treatment

A calorimetric procedure was used to determine the effective ultrasound power (P, expressed as W) transferred into the medium for each treatment condition. The ultrasound probe and the bath densities (UD) were expressed as watts per unit volume of the sonicated solution (W/L) according to UD = P/V and performed using a Scientz-IID generator (Ningbo Scientz Biotechnology Co., Ningbo, China).

A full factorial design was employed to evaluate the ultrasound treatment on anthocyanin extraction. Under a general degradative setting condition, the effects of the parameters, including ultrasound time (5, 10, 20, 30, 40, and 50 min), temperature (10, 25, and 40 °C) and solvent (100% (pure) MeOH, 70% *v/v* (aqueous) MeOH, 100% (pure) EtOH, and 70% *v/v* (aqueous) EtOH) were systematically investigated. The general degradative condition of all treatments was investigated using the following parameters: pH 2, UD 135 W/L, solid–liquid ratio of 1:30 g/mL. Specifically, the pH of the solution was measured by using a pH meter with an accuracy of 0.05 pH units, and the initial pH value of solution was adjusted by 0.1 M HCl. Then, 0.5 g of blueberry pomace power was added to a glass tube and then mixed with 15 mL of solvent before exposing to ultrasound. The temperatures during the extractions were evaluated with a digital thermometer and were held constantly at a desired value by a thermostatic water bath. Throughout the ultrasound process, the tube was sealed and fixed. Meanwhile, the sample with the same solvent at the same temperature was macerated for 3 h as the control.

### 4.5. Conventional Solvent Extraction Treatment

Blueberry pomace power (0.5 g) was placed in a glass tube and then mixed with 15 mL of solvent by using a 20 mm rotor magnetic stirrer at a constant 25 °C. The TA content in blueberry pomace was quantified after extraction for 1, 2, 3, 5, and 7 h. The time at which the TA reached an unchanged quantity by conventional solvent extraction was defined as the control and was compared with the UAE method.

### 4.6. Determination of Total and Individual Anthocyanins Contents

The TA content in the prepared extracts was estimated using the pH-differential method [32]. KCl buffer (0.025 M, pH 1.0) and NaOAc buffer (0.4 M, pH 4.5) were prepared with pH adjustment using concentrated HCl. Briefly, samples were prepared using the buffers, thus the absorbance at 520 nm would fall within 0.1–1.4 AU while not exceeding the 1:5 sample/buffer ratio, and the absorbance of each dilution was measured at 520 and 700 nm. TA contents in the extracts were calculated and expressed as cyanidin-3-*O*-glucoside (C3G) equivalents according to the following formula:

(1) $$TA\;\left(mg/g\right)=\frac{\left(A\;\times Mw\;\times DF\;\times V\;\times 1000\right)}{Ma\;\times L\times m}$$

where A (absorbance) = (A_520_ nm pH 1.0 - A_700_ nm pH 1.0) - (A_520_ nm pH 4.5 - A_700_ nm pH 4.5), Mw (molecular weight) = 449.2 g/mol, DF = dilution factor, Ma (extinction coefficient) = 26,9000 mol/L/cm, L (path length) = 1 cm, V = the total volume (mL), m = the quantity of blueberry pomace (g), and a multiplication factor of 1000 was used to convert from grams to milligrams. Results were defined as milligrams of C3G equivalents per gram of blueberry pomace.

The IA content in the prepared extracts was estimated by HPLC as reported elsewhere [24] with modifications. Briefly, the Agilent 1100 HPLC system (Agilent Technologies) used was equipped with an autosampler module, a degasser, a binary pump, a column heater/selector, a UV–visible-diode array detector, and a Zorbax column (4.6 × 250 mm, 5 μm; Agilent Technologies Co., Ltd.). The analysis was undertaken at a constant temperature of 35 °C and a flow rate of 0.6 mL/min. Eluent A was 6% *v/v* aqueous formic acid, and eluent B was 6% *v/v* ACN–formic acid. The gradient program was as follows: 85–70% B (0–20 min), 70–65% B (20–25 min), 65–85% B (25–30 min), and 85% B (30–35 min). Simultaneous monitoring was performed at 520 nm.

### 4.7. Statistical Analysis and Figure Plotting

All experiments were performed in triplicate, and the results were expressed as mean ± standard deviation. One-way analysis of variance (ANOVA; *p* < 0.05) and Duncan’s multiple range test were performed using SPSS 19.0 (SPSS, Inc., Chicago, IL, USA). The main effects of each factor and their interactions were analyzed by a GLM univariate method. All figures were exported using Origin Software (OriginLab Corp., Northampton, MA, USA). 

## 5. Conclusions

In this work, the anthocyanin composition of blueberry pomace was characterized by LC–MS, and malvidin-3-galactoside, which represented the most abundant IA, was selected as the target IA analyte. A GLM univariate methodology and all factor models were successfully employed to compare and optimize the parameters for TA and IA extraction. In comparison to a conventional solvent extraction method, UAE resulted in higher recoveries of both TA and IA. Moreover, IA tended to undergo more degradation compared with TA during UAE. The study indicated that ultrasound is a promising technology in the extraction of desired bioactive components from food industry residues, while harsh conditions should be avoided to prevent the degradation of a target compound. A specific extraction process of IA preparation is of more value and significance for monomer preparation than TA.

## Figures and Tables

**Table 1:** Identification of anthocyanins in blueberry pomace using liquid chromatography-mass spectrometry (LC–MS).

Peak	Retention Time	+ESIMS (m/z)	Formula	Proportion (%)	Tentative Identification
1	12.109	429	-	15.29	unknown
2	13.327	465	C_21_H_21_O_12_	7.00	delphinidin-3-galactoside
34	15.055	384	-	2.27	unknown
	15.521	465	C_21_H_21_O_12_	9.84	delphinidin-3-glucoside
5	16.618	464	-	1.29	unknown
6	17.412	435	C_20_H_19_O_11_	9.46	delphinidin-3-arabinoside
7	18.848	449	C_21_H_21_O_11_	8.01	cyanidin-3- glucoside
8	20.576	479	C_22_H_23_O_12_	0.99	petunidin-3-galactoside
9	21.116	479	C_22_H_23_O_12_	5.50	petunidin-3-glucoside
10	22.362	493	C_23_H_25_O_12_	18.56	malvidin-3-galactoside
11	23.799	449	C_21_H_21_O_11_	11.11	petunidin-3-arabinoside
12	24.33	493	C_23_H_25_O_12_	9.15	malvidin-3- glucoside
13	26.076	463	C_22_H_23_O_11_	0.12	peonidin-3-galactoside

## Supplementary Materials

The following are available online. Figure S1: The effect of time on total anthocyanin extracted by conventional solvent extraction method. Different letters on bars show significant differences (*p* < 0.05), Figure S2: Effect of time on the stability of total anthocyanins in blueberry pomace under ultrasound treatment and conventional solvent extraction. Different letters on bars show significant differences (*p* < 0.05), Figure S3: Effect of temperature on the stability of total anthocyanins in blueberry pomace under ultrasound treatment and conventional solvent extraction. Different letters on bars show significant differences (*p* < 0.05), Figure S4: Effect of solvent on the stability of total anthocyanins in blueberry pomace under ultrasound treatment and conventional solvent extraction. Different letters on bars show significant differences (*p* < 0.05), Figure S5: Effect of interaction on total anthocyanins from blueberry pomace during ultrasound extraction course, Table S1: Effect of time on the stability of total anthocyanins in blueberry pomace under ultrasound treatment and conventional solvent extraction, Table S2: Effect of time on the stability of individual anthocyanin in blueberry pomace under ultrasound treatment and conventional solvent extraction.
