# Supplementary material for: Ultrasound Treatment on Stability of Total and Individual Anthocyanin Extraction from Blueberry Pomace: Optimization and Comparison
Source: Molecules. 2019 Jul 18;24(14):2621. doi: 10.3390/molecules24142621 (PMC6680923; doi:10.3390/molecules24142621)
Supplement: Supplementary file 1 [file molecules-24-02621-s001.pdf]

molecules

Supplementary File

Ultrasound Treatment on Stability of Total and Individual Anthocyanin Extraction from Blueberry Pomace: Optimization and Comparison

Weiwei Hu †, Hui Gong †, Lanqi Li, Shiguo Chen and Xingqian Ye *

Zhejiang Key Laboratory for Agro-Food Processing, Fuli Institute of Food Science, College of Biosystem Engineering and Food Science, Zhejiang University, Hangzhou 310058, China

* Correspondence: psu@zju.edu.cn (X.Y.); +86-571-8898-2155 (X.Y.)

† These authors contributed equally to this work

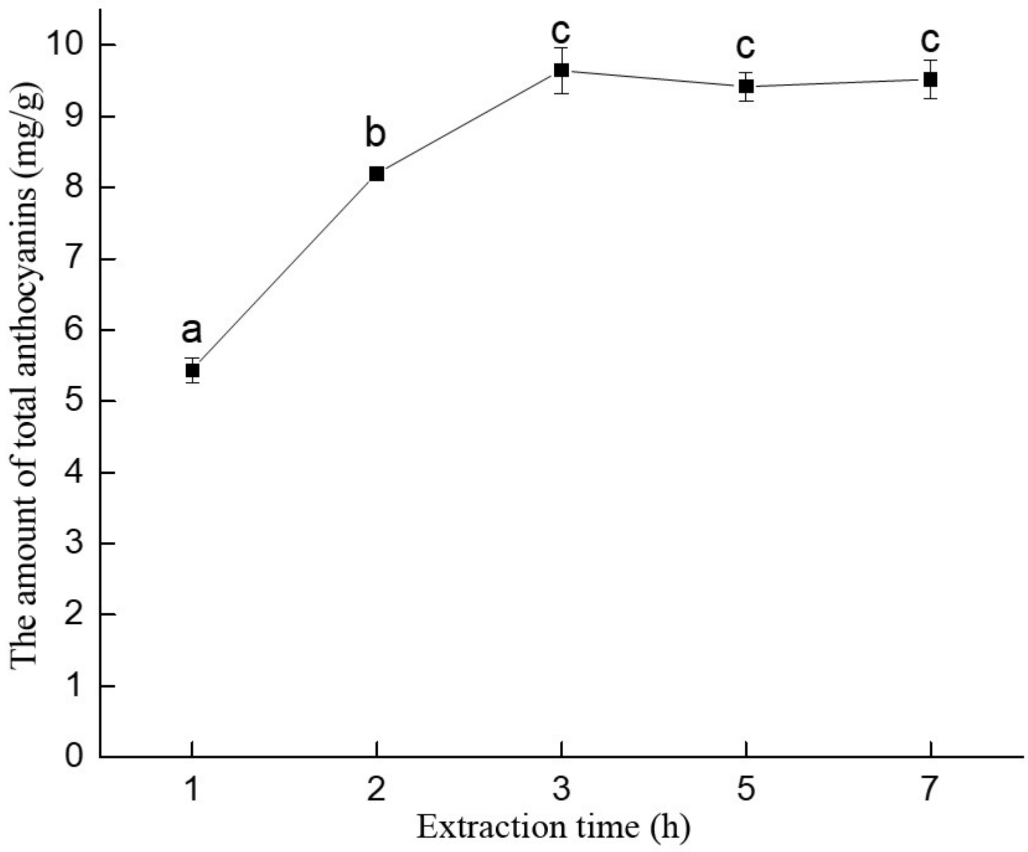

Figure 1. The effect of time on total anthocyanin extracted by conventional solvent extraction method. Different letters on bars show significant differences ($p < 0.05$).

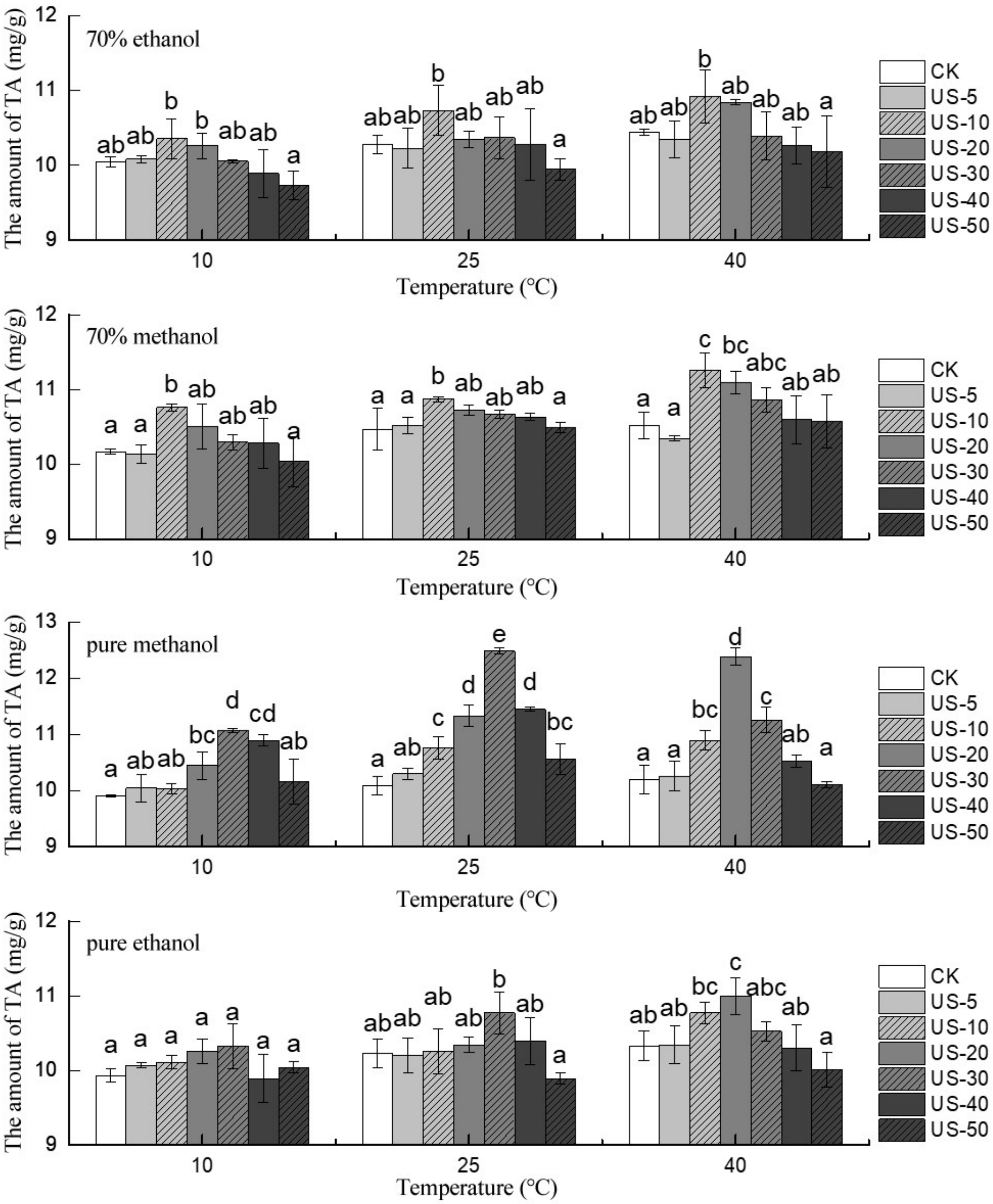

Figure 2. Effect of time on the stability of total anthocyanins in blueberry pomace under ultrasound treatment and conventional solvent extraction. Different letters on bars show significant differences ($p < 0.05$).

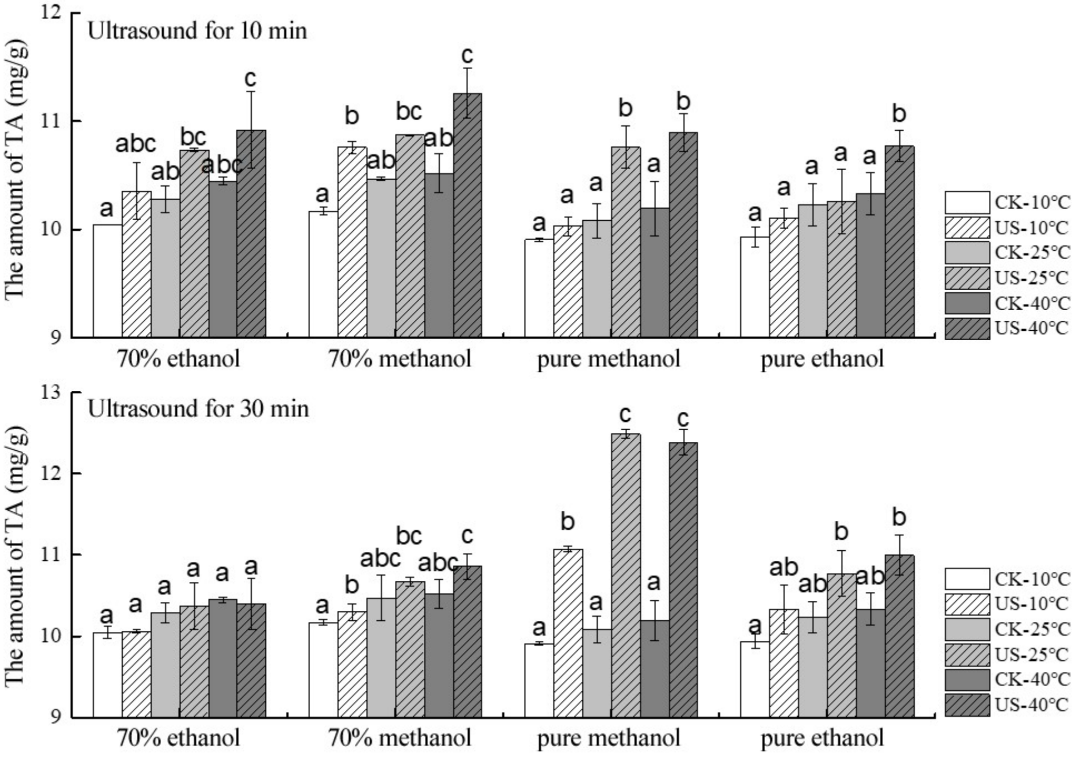

Figure 3. Effect of temperature on the stability of total anthocyanins in blueberry pomace under ultrasound treatment and conventional solvent extraction. Different letters on bars show significant differences ($p < 0.05$).

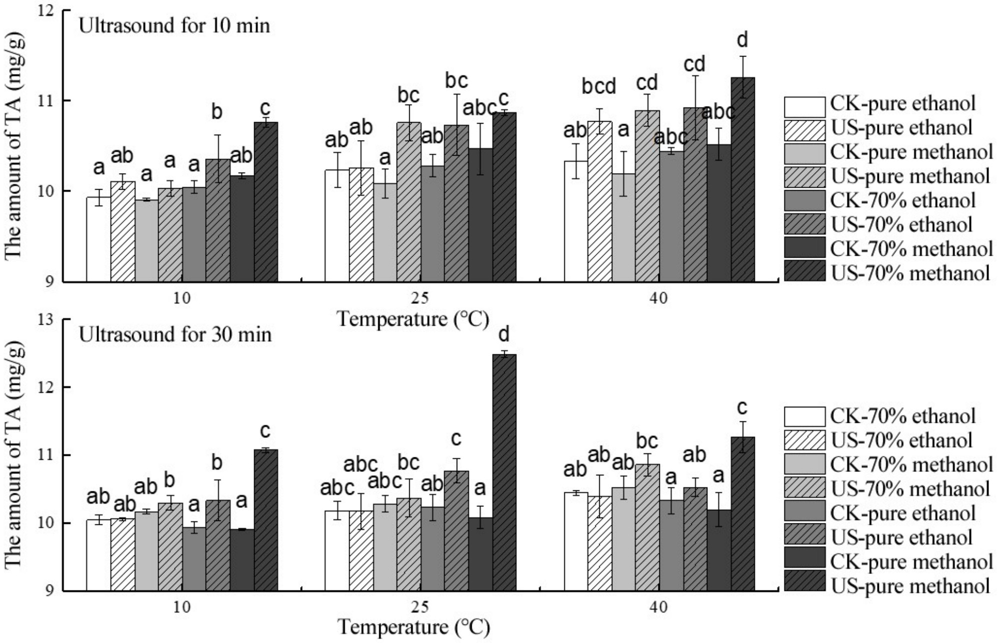

Figure S4 Effect of temperature on the stability of total anthocyanins in blueberry pomace under ultrasound treatment and conventional solvent extraction. Different letters on bars show significant differences ($p < 0.05$).

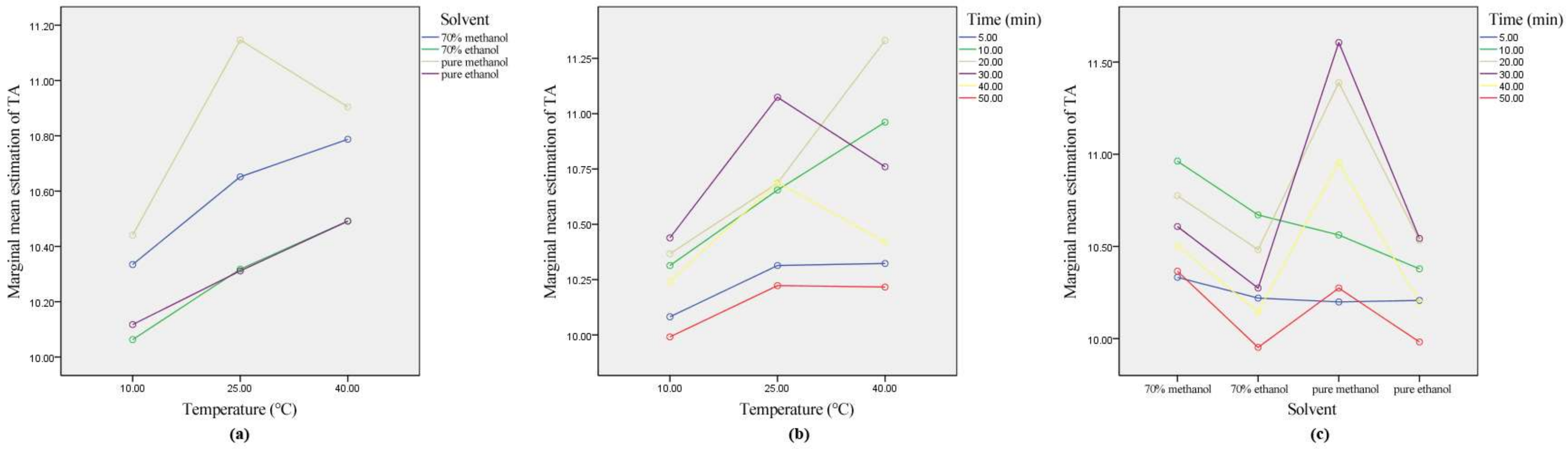

Figure 5. Effect of interaction on individual anthocyanins from blueberry pomace during ultrasound extraction course. Letters (**a**–**c**) represent interactions of solvent–temperature, temperature–time and solvent–time.

Table S1. Effect of time on the stability of total anthocyanins in blueberry pomace under ultrasound treatment and conventional solvent extraction.

Sample	Temp (℃)	CK (mg/g)	US-5min (mg/g)	US-10 min (mg/g)	US-20 min (mg/g)	US-30 min (mg/g)	US-40 min (mg/g)	US-50 min (mg/g)
70% ethanol	10	10.04 ± 0.07	10.08 ± 0.05	10.36 ± 0.27	10.26 ± 0.17	10.06 ± 0.02	9.89 ± 0.32	9.73 ± 0.19
	25	10.28 ± 0.12	10.23 ± 0.27	10.73 ± 0.34	10.34 ± 0.11	10.37 ± 0.28	10.28 ± 0.48	9.94 ± 0.14
	40	10.45 ± 0.04	10.34 ± 0.25	10.92 ± 0.35	10.85 ± 0.04	10.40 ± 0.32	10.26 ± 0.24	10.18 ± 0.48
70% methanol	10	10.17 ± 0.04	10.13 ± 0.12	10.76 ± 0.05	10.51 ± 0.30	10.29 ± 0.11	10.28 ± 0.34	10.03 ± 0.34
	25	10.47 ± 0.28	10.52 ± 0.11	10.87 ± 0.04	10.72 ± 0.07	10.67 ± 0.06	10.63 ± 0.05	10.50 ± 0.07
	40	10.52 ± 0.18	10.34 ± 0.04	11.26 ± 0.23	11.10 ± 0.15	10.86 ± 0.16	10.60 ± 0.32	10.57 ± 0.35
pure methanol	10	9.91 ± 0.02	10.04 ± 0.25	10.03 ± 0.09	10.45 ± 0.25	11.07 ± 0.04	10.90 ± 0.11	10.16 ± 0.41
	25	10.08 ± 0.16	10.29 ± 0.11	10.76 ± 0.19	11.33 ± 0.19	12.49 ± 0.05	11.45 ± 0.04	10.56 ± 0.27
	40	10.19±0.25	10.26 ± 0.27	10.90 ± 0.18	12.39 ± 0.16	11.26 ± 0.23	10.52 ± 0.11	10.11 ± 0.05
pure ethanol	10	9.93 ± 0.09	10.07 ± 0.04	10.11 ± 0.09	10.26 ± 0.17	10.33 ± 0.30	9.89 ± 0.32	10.04 ± 0.07
	25	10.23 ± 0.19	10.21 ± 0.23	10.26 ± 0.30	10.34±0.11	10.77±0.28	10.40±0.32	9.89±0.07
	40	10.33 ± 0.19	10.34 ± 0.25	10.77 ± 0.14	11.00±0.25	10.53±0.13	10.30±0.31	10.01±0.23

Table S2. Effect of time on the stability of individual anthocyanin in blueberry pomace under ultrasound treatment and conventional solvent extraction.

Sample	Temp (℃)	CK (mg/g)	US-5min (mg/g)	US-10min (mg/g)	US-20min (mg/g)	US-30min (mg/g)	US-40min (mg/g)	US-50min (mg/g)
70% ethanol	10	2.55±0.14	2.48±0.07	2.50±0.20	2.68±0.05	2.93±0.04	2.85±0.08	2.82±0.05
	25	2.62±0.01	2.51±0.04	2.63±0.07	2.85±0.01	3.02±0.03	2.95±0.11	2.79±0.03
	40	2.74±0.01	2.55±0.15	2.78±0.01	3.10±0.05	3.18±0.23	3.57±0.02	3.36±0.05
70% methanol	10	2.40±0.05	2.14±0.07	2.35±0.01	2.51±0.08	2.68±0.07	2.35±0.11	2.33±0.20
	25	2.46±0.03	2.10±0.14	2.31±0.12	2.46±0.02	2.69±0.06	2.45±0.13	2.34±0.01
	40	2.50±0.02	2.35±0.06	2.42±0.04	2.62±0.02	2.81±0.08	2.78±0.06	2.72±0.10
pure methanol	10	2.36±0.12	2.05±0.05	2.27±0.11	2.38±0.09	2.63±0.01	2.31±0.17	2.15±0.07
	25	2.44±0.11	2.27±0.11	2.29±0.14	2.46±0.02	2.65±0.00	2.32±0.06	2.34±0.03
	40	2.57±0.07	2.35±0.06	2.42±0.04	2.62±0.02	2.69±0.13	2.87±0.07	2.71±0.10
pure ethanol	10	2.23±0.05	1.97±0.05	2.27±0.11	2.33±0.20	2.51±0.08	2.42±0.03	2.39±0.18
	25	2.25±0.09	2.06±0.20	2.35±0.18	2.50±0.21	2.65±0.00	2.45±0.12	2.43±0.11
	40	2.29±0.16	2.09±0.18	2.18±0.05	2.45±0.10	2.72±0.09	2.53±0.07	2.49±0.14
